# A Liposomal Delivery System of Blueberry Anthocyanins Ameliorates Corneal Laser Injury

**DOI:** 10.3390/biom16050703

**Published:** 2026-05-11

**Authors:** Zihan Lv, Chaoran Li, Di Liang, Guangrui Chen, Mengqi Qiu, Zhiyun Meng, Ruolan Gu, Hui Gan, Zhuona Wu, Zaifu Yang, Guifang Dou

**Affiliations:** Academy of Military Medical Sciences, Beijing 100850, China; 15081156078@163.com (Z.L.); lichaoran0922@163.com (C.L.); liangdi0807@163.com (D.L.); chen_guangrui@163.com (G.C.); aqmq5213@163.com (M.Q.); mengzhiyun@vip.163.com (Z.M.); guruolan@bmi.ac.cn (R.G.); ganh2003@163.com (H.G.); wznphd@126.com (Z.W.)

**Keywords:** blueberry anthocyanins, nanostructures, liposomes, corneal laser damage

## Abstract

This study aims to develop and systematically evaluate a new lipid-based formulation of blueberry anthocyanins, which can accelerate the healing effect of the cornea. The study first successfully screened and optimized the formulation and preparation process for blueberry anthocyanin liposomes. Characterization via transmission electron microscopy and dynamic light scattering revealed uniformly distributed, near-spherical liposomes with distinct phospholipid bilayers. Key physicochemical parameters—particle size, zeta potential, encapsulation efficiency, and drug loading capacity—all met formulation standards. In vivo pharmacodynamic experiments demonstrated that topical administration of blueberry anthocyanin liposomes significantly accelerated the repair process and effectively mitigated depressional damage to the corneal epithelium in a New Zealand white rabbit corneal injury model induced by 10.6 μm mid-infrared CO_2_ laser. In summary, the blueberry anthocyanin liposomes successfully prepared in this study exhibit excellent performance, effectively enhancing drug exposure levels in vivo and promoting corneal repair. This provides reliable experimental evidence for the development of plant natural active ingredients in ophthalmic treatments.

## 1. Introduction

In military and industrial settings, corneal injuries caused by laser weapons or accidental laser exposure represent a growing public health and safety concern [1]. Lasers can damage corneal tissue through multiple pathways, including thermal effects, photochemical effects, and acoustic shock waves, leading to epithelial defects, stromal edema, and intense oxidative stress and inflammatory responses [2]. If not repaired promptly, this can severely impair vision [3]. Therefore, developing therapeutic strategies that effectively promote corneal repair and mitigate oxidative and inflammatory damage is crucial.

Natural product blueberry anthocyanins demonstrate significant potential in this field. Blueberry anthocyanins are a class of water-soluble flavonoid polyphenolic compounds widely present in blueberries, garnering significant attention for their potent antioxidant and anti-inflammatory activities [4]. Research indicates these properties confer substantial potential for protecting ocular health. Studies confirm their exceptional antioxidant capacity, effectively scavenging free radicals such as hydroxyl radicals and superoxide anions [5]. More importantly, multiple animal studies have revealed their protective effects against light-induced ocular damage. Research indicates that blueberry extracts significantly improve retinal tissue structure in light-damaged mice, inhibit lipid peroxidation within the retina, and enhance the activity of antioxidant enzymes such as superoxide dismutase (SOD) and glutathione peroxidase (GSH-Px) [6]. Another rat study further confirmed that blueberry anthocyanins effectively prevent thinning of the outer nuclear layer of the retina [7], with their protective mechanism closely linked to their anti-lipid peroxidation effects [8]. However, the inherent chemical instability of blueberry anthocyanins severely limits their biological effects. They are highly sensitive to environmental factors such as pH, light, temperature, and oxygen, and are prone to degradation and inactivation during processing, storage, and passage through the gastrointestinal tract [9]. More critically, even when stable, unmodified blueberry anthocyanin extracts face the challenge of low bioavailability, primarily due to poor membrane permeability and extensive metabolism within the body [10]. These limitations collectively result in extremely low effective concentrations reaching target tissues after oral administration, severely restricting their application in functional foods and pharmaceuticals. Currently, conventional formulations of blueberry anthocyanins are often simple extract solutions. This form is easily degraded by the gastrointestinal environment after oral administration, resulting in extremely low bioavailability. When used for topical ocular administration, factors such as the corneal barrier and tear flushing similarly hinder the achievement and maintenance of effective therapeutic concentrations at the target site [11]. Currently, clinical treatment options for corneal laser injury are extremely limited, primarily focusing on infection prevention and inflammation relief, with a lack of specific therapies that can efficiently promote tissue regeneration and functional recovery [12]. Blueberry anthocyanins possess potent antioxidant (e.g., via activation of the Nrf2 pathway) and anti-inflammatory (e.g., via inhibition of the NF-κB pathway) properties. Therefore, developing novel delivery systems to overcome these bottlenecks is crucial for realizing the therapeutic potential of blueberry anthocyanins [13,14].

Among numerous candidate strategies, liposome technology demonstrates significant advantages [15]. Liposomes are hollow vesicles composed of phospholipid bilayers, whose unique structure enables the encapsulation of both hydrophilic and hydrophobic drugs simultaneously [16]. Encapsulating bioactive substances within liposomes creates a microenvironment isolated from external conditions [17], effectively shielding them from adverse effects of light, oxygen, and pH fluctuations, thereby significantly enhancing stability [18]. Furthermore, as delivery carriers, liposomes facilitate the crossing of biological barriers by enhancing cellular uptake and internalization processes, after encapsulation in liposomes, the phospholipid bilayer isolates the anthocyanins from the external environment, protecting them from degradation during storage and administration [19]. In lipid-based topical ophthalmic formulations—which serve as liposomal systems for delivering the bioactive components of blueberry extract—this protective effect not only extends the formulation’s shelf life but, more importantly, ensures that the drug remains active and effective upon reaching the ocular surface. Furthermore, liposomes reduce direct contact between the drug and ocular surface tissues, thereby mitigating the toxic effects of irritating drugs on the cornea and conjunctiva and improving treatment safety. For chronic eye diseases requiring long-term medication, this advantage holds significant clinical importance. Traditional blueberry anthocyanin solutions exhibit a typical “pulse-like” release profile after administration: a high initial concentration rapidly declines, with a short duration of action, and most of the drug is washed away by tears. This rapid clearance makes it difficult for anthocyanins to reach effective concentrations in the target ocular tissues. Liposomes, however, enable sustained drug release through the gradual degradation of the phospholipid bilayer and the stepwise diffusion of the drug from the vesicles.

As the outermost layer of the eye, the cornea can be damaged by trauma, infection, chemical irritation, dry eye syndrome, improper contact lens use, and corneal dystrophy, among other causes [20]. Corneal damage can be treated with medication, protective eyewear, artificial tears, corneal repair surgery, and avoidance of irritants [21]. Blueberry anthocyanins inherently counteract photo-oxidative damage by enhancing antioxidant enzyme activities (e.g., SOD, GSH-Px) and reducing malondialdehyde (MDA) levels. Theoretically, this makes them ideal candidate drugs for treating such injuries [7,22]. However, due to its poor stability and difficulty in effectively penetrating the corneal barrier, direct application of blueberry extract yields minimal efficacy. Molecular modifications such as acetylation enhance the intrinsic stability of anthocyanins at the chemical structural level; auxochrome effects and metal chelation enhance their stability through intermolecular non-covalent interactions; while encapsulation delivery systems provide protection through physical barriers. In practical applications, combining multiple strategies often yields superior stabilization results. This study employs nanoliposome encapsulation technology as a delivery vehicle for blueberry anthocyanins, precisely because of its dual advantages of protecting anthocyanins from degradation and prolonging their retention time in the eye, thereby enabling their therapeutic effects to be more fully realized.

In summary, this study aims to develop a novel blueberry anthocyanin delivery system based on liposome technology to address its core issues of low stability and delivery efficiency. Furthermore, it explores its potential application in repairing corneal laser damage, offering a new strategy to tackle this clinical challenge.

## 2. Materials and Methods

### 2.1. Materials

Blue Beauty No. 1 Powder (Blueberry extract, D-B-LYLmAn2020-007) was purchased from Zhejiang Blue Beauty Company; Soy Lecithin (C16452900) from Shanghai Yuanye Biotechnology Co., Ltd. (Shanghai, China); β-Sitosterol (O21IS228709) from Shanghai Yuanye Biotechnology Co., Ltd. (Shanghai, China); Chloromethane (20240422) from Sinopharm Chemical Reagent Co., Ltd. (Shanghai, China); Pe-tunidin-3-O-arabinopyranoside (ZT-20992) from Shanghai zzstandards (Shanghai, China); Mal-vidin-3-O-arabinopyranoside (ZT-21044) from Shanghai zzstandards; Del-phinidin-3-O-Arabinoside (ZT-21020) from Shanghai zzstandards; Petunidin-3-O-Glucoside (ZT-60581) from Shanghai zzstandards; Malvidin-3-O-Glucoside (ZES-0911S) from France extra-synthese (Genay, France); Digitalis glycoside-glucoside (ZES-0938S) from France extrasynthese; Pepsin (H2329190) from Shanghai Aladdin Biochemical Technology Co., Ltd. (Shanghai, China); and Trypsin (J2430530) from Shanghai Aladdin Biochemical Technology Co., Ltd. (Shanghai, China) All supplementary reagents were of analytical grade and procured from Sinopharm Chemical Reagent Co. Ltd., Shanghai, China. Ultra-pure Milli-Q water, produced by Millipore (Burlington, MA, USA), was utilized throughout the study.

### 2.2. Animals

Fifteen male New Zealand White rabbits weighing 2.5 kg were purchased from Beijing Keyu Laboratory Animal Center, with animal certification number SYXK (Military) 2025-004. They were housed in climate-controlled facilities (22 ± 2 °C, 46 ± 20% relative humidity, 12 h light/dark cycle) for one week of acclimatization. Animals were randomly assigned to the following groups: control group, model group (model), positive drug group (VA), blueberry anthocyanin aqueous solution group (ANC-aq), and blueberry anthocyanin liposome group (ANC-LP). In each rabbit, only the laser-injured eye was treated with 50 μL of the formulation once daily for 14 consecutive days. The concentration of both ANC-aq and ANC-LP is 2 mg/mL. Each experimental group consisted of 3 rabbits (*n* = 3). Before each administration, the ANC-LP formulation was sterilized by ^60^Co irradiation to ensure its sterility. Prior to the experiment, the animals’ eyes were examined using a slit-lamp biomicroscope to ensure clarity of the anterior segment refractive media.

### 2.3. Methods

#### 2.3.1. Synthesis of ANC-LP

Accurately weighed 0.0107 g of cholesterol and 0.0503 g of soybean lecithin, and they were dissolved in 15 mL of chloroform, followed by sonication for 10 min until completely dissolved. Accurately weighed 0.0105 g of blueberry anthocyanin extract, which was dissolved in 10 mL of ultrapure water and sonicated for 40 min until completely dissolved. Under high-speed magnetic stirring at 450 rpm, the blueberry anthocyanin aqueous solution was slowly injected into the organic phase using a 2 mL syringe to form a purple emulsion. The emulsion was transferred to a 100 mL rotary evaporator flask, and the organic solvent was removed by rotary evaporation under reduced pressure at 37 °C and 80 rpm. Subsequently, 10 mL of ultrapure water was added to the flask, and rotary hydration was continued for 2 h. The suspension was then transferred to a 15 mL centrifuge tube and sonicated with a probe in an ice-water bath (8 s on, 2 s off, 500 W power, repeated 70 times) to ensure uniform dispersion, yielding blueberry anthocyanin liposomes (ANC-LP).

#### 2.3.2. Morphological Observations

The morphology of ANC-LP was observed using a transmission electron microscope (JEM-2100Plus, JEOL, Tokyo, Japan) with an electron beam voltage of 80 kV. An ultrathin carbon film was selected and placed face-up on clean filter paper. An appropriate amount of sample was taken and added to a small beaker containing distilled water, followed by ultrasonic dispersion for 15 min. A glass capillary tube was used to draw up the mixture and dispense it onto the carbon film. After the solvent had evaporated, the sample was loaded onto a sample holder. Under vacuum suction, the sample holder was slowly slid into the electron microscope. Then, focusing, image capturing, and saving were performed.

#### 2.3.3. Size and Zeta Potential

The particle size and zeta potential of ANC-LP were measured using a Malvern laser particle size analyzer (Zetasizer Nano ZS, Malvern Instruments Ltd., Malvern, UK). One milliliter of the ANC-LP sample resuspended in distilled water was taken and transferred to a cuvette. A dynamic light scattering (DLS) instrument was used to measure its average particle size, polydispersity index (PDI), and zeta potential. The measurement temperature was set to 25 °C, and the measurement was repeated three times for each sample.

#### 2.3.4. Determination of Encapsulation Efficiency and Drug Yield

The ANC-LP suspension was centrifuged at 3000 rpm for 30 min to remove unencapsulated blueberry anthocyanin extract. After centrifugation, the pellet (liposomes) and the supernatant (free drug) were collected separately. To isolate the blueberry extract, liquid chromatography and mass spectrometry conditions were established using a UV-visible spectrophotometer (UV-2600, Shimadzu, Kyoto, Japan) and UPLC-MS/MS (AB SCIEX Triple Quad 6500^+^, Framingham, MA, USA). A standard curve was plotted to determine the blueberry anthocyanin content in the sample. Chromatograph: Exion TRIPLE QUAD 6500^+^; Column: Waters ACQUITY UPLC-BEH C18 (2.1 mm × 50 mm, 1.7 μm); Column temperature: 35 °C; Flow rate: 0.3 mL/min; Analysis time: 4 min; Injection volume: 1 μL; Needle flush solution: methanol: isopropanol: water 2:2:1; Mobile phase: Phase A: 0.2% formic acid in water; Phase B: 0.1% formic acid in acetonitrile.

The mobile phase gradient elution conditions are as follows: 0–0.50 min, maintain 95% Phase A and 5% Phase B; 0.50–1.00 min, Phase A linearly decreases from 95% to 10%, and Phase B increases from 5% to 90%; 1.00–2.50 min, maintain 10% Phase A and 90% Phase B; 2.50–4.00 min: Phase A increases from 10% back to 95%, and Phase B decreases from 90% to 5%, maintaining this ratio until 4.00 min.

Mass spectrometry detection uses an electrospray ionization (ESI) source in positive ion mode, with an acquisition time of 4 min. Key mass spectrometry parameters are set as follows: cone voltage 60 V, collision energy 20 eV, capillary voltage 3.00 kV; cone flow rate 150 L/h, source temperature 150 °C, desolvation temperature 350 °C, desolvation flow rate 650 L/h, collision flow rate 0.15 mL/min, and atomizer pressure 6.0 bar. This method has been validated and demonstrates good specificity, precision, accuracy, and stability, making it suitable for the determination of anthocyanin content in blueberries. Each liposome was spiked with an internal standard and diluted with acetonitrile. The samples were analyzed alongside freshly prepared standards and quality control standards.

$$EE\left(\%\right)=\frac{C_{loaded}}{C_{total}}\times 100\%$$

$$DL\left(\%\right)=\frac{C_{loaded}}{C_{lipid}}\times 100\%$$

#### 2.3.5. pH and Osmolarity

The pH of the ANC-LP formulation was determined using a calibrated pH meter (PB-10, Sartorius, Goettingen, Germany) at room temperature by directly inserting the electrode into the liposome sample. Measurements were performed in triplicate. The osmometer (Osmo210, YASN, London, UK) was calibrated using 50 mOsm/kg and 850 mOsm/kg calibration solutions. Then, a 290 mOsm/kg reference solution was tested to determine whether the instrument calibration met operational requirements. Using a 100 µL pipette, 50 µL of the sample was transferred to the sample tube, ensuring that the sample was added to the bottom of the tube without any air bubbles. The test was then performed.

#### 2.3.6. Establishment of a Corneal Laser Injury Model

Thirty minutes prior to the experiment, the pupils were dilated three times using compound tropicamide eye drops (5 min intervals) to achieve full pupil dilation in rabbits. Five minutes before the experiment, the corneal surface was anesthetized with procaine hydrochloride eye drops. The animal was secured, and the eye’s position and angle were precisely adjusted to ensure that the laser beam targeted the corneal center. A 10.6 μm CO_2_ laser was used to ablate the rabbit cornea under the following conditions: exposure time 0.1 s, spot diameter 3 mm, power 20 W. Each eye received a single laser spot, positioned as close as possible to the corneal center [23]. All experiments were carried out in compliance with the ethical principles governing the welfare of experimental animals and received approval from the Ethics Committee of the Beijing Institute of Radiological Medicine (No. IACUC-DWZX-2024-P626, 4 March 2024).

#### 2.3.7. Slit-Lamp Microscopy Examination of Damaged Corneas

Corneal damage was observed using a slit-lamp microscope immediately before laser injury and at 30 min, 1 day, 3 days, 5 days, 7 days, 10 days, and 14 days post-injury. Observation methods included wide-field oblique illumination and narrow-slit illumination to assess corneal surface morphology and injury depth, with photographic documentation. The anterior segment was dilated prior to observation.

#### 2.3.8. HD-OCT Observation Methods for Damaged Corneas

Cirrus High-Definition Optical Coherence Tomography (HD-OCT) was used to perform cross-sectional scans of the corneal injury site at 30 min, 1 day, 3 days, 5 days, 7 days, 10 days, and 14 days post-injury. Images were captured near the center of the injury patch, with at least three scans performed at each location to ensure the clearest possible image quality. The corneal epithelium and stroma were manually segmented based on image grayscale. Corneal thickness at the lesion center was measured using the built-in ruler of the Cirrus HD-OCT system for statistical analysis.

#### 2.3.9. Histopathological Observation Methods for Damaged Corneas

Fourteen days after laser injury, New Zealand White rabbits in each group were euthanized by air embolism, and their eyeballs were removed. After washing the external surface of the eyeballs with physiological saline to remove bloodstains, the entire eyeballs were promptly immersed in FAS eyeball fixative (Servicebio) for 4 h. Corneas underwent graded dehydration and clearing, followed by paraffin embedding and sectioning. Sections were stained with hematoxylin and eosin (H&E) and sealed with neutral resin. Histopathological morphology was observed under an optical microscope and imaged using a slide scanning system.

#### 2.3.10. Detection of Inflammatory Cytokines in Plasma

Blood was drawn from the rabbit ear vein. The sample was centrifuged at 3500 rpm for 15 min, and the supernatant plasma was set aside. Rabbit plasma samples were stored at −80 °C to maintain their integrity. Subsequently, cytokine concentrations in these samples were measured using an ELISA kit (Cusabio, Wuhan, China) in strict accordance with the manufacturer’s guidelines. Absorbance readings were taken at 450 nm for each well. The levels of inflammatory cytokines were then accurately calculated using a standard curve.

#### 2.3.11. Statistical Analysis

Statistical analysis of the measured data was performed using SPSS 23.0 software, with data expressed as x ± SD. Paired *t*-tests were used to compare whether there were significant differences in thickness at various time points post-injury compared to pre-injury levels (*p* < 0.05).

## 3. Results

### 3.1. Macroscopic Appearance of ANC-LP

**Figure 1:**
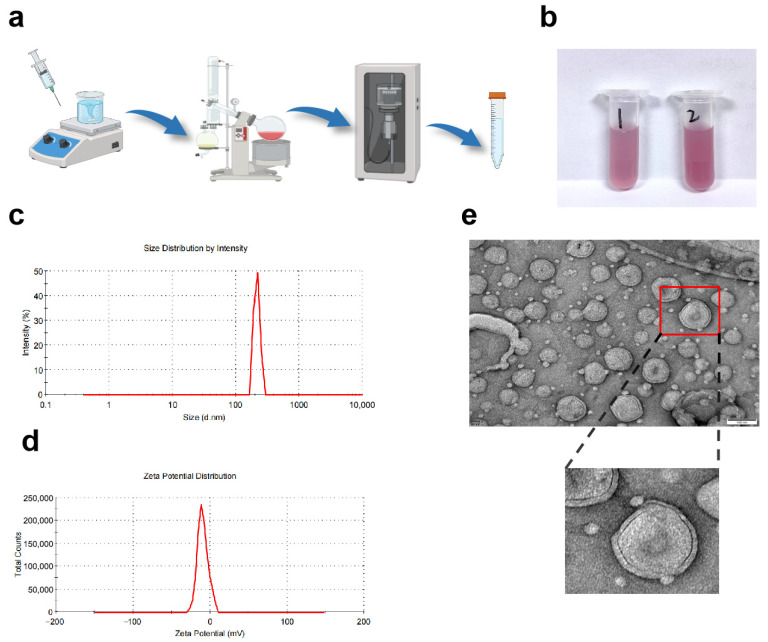
ANC-LP Preparation Process and Characterization Results. (**a**) Experimental steps from solution mixing and stirring → rotary evaporation concentration → ultrasonic probe treatment → final sample collection. (**b**) The prepared sample is a purple-red liquid. Sample 1 is ANC-LP filtered through a 0.22 μm microporous membrane, while Sample 2 is unfiltered ANC-LP. Membrane filtration removes larger particles, resulting in a clearer solution compared to the unfiltered sample. (**c**) Dynamic light scattering particle size distribution measured by Malvern laser particle size analyzer: The peak is centered at 248.9 ± 21.5nm, indicating good particle size dispersion of the product. (**d**) Zeta potential reflects surface charge, with a result of −9.14 ± 0.54 mV, indicating ANC-LP carries a negative charge and exhibits good stability. (**e**) Transmission electron microscopy (TEM) reveals the microscopic morphology of the particles: they are spherical in shape with good dispersion, and the spherical structure of individual particles can be observed. Scale bar = 100 nm.

This experiment achieved the construction of ANC-LP through a stepwise process involving “solution stirring and mixing–rotary evaporation concentration–ultrasonic dispersion.” This preparation workflow offers strong controllability and excellent process reproducibility, enabling stable, efficient encapsulation of anthocyanins and uniform preparation of liposomes. Figure 1b shows the prepared ANC-LP as a uniform, transparent, purplish-red dispersion. No significant color differences, phase separation, or precipitation were observed between the two samples. This appearance indicates excellent dispersion homogeneity within the liposome system, confirming effective encapsulation and uniform distribution of anthocyanins within the carrier. No phase separation or aggregation of active components occurred.

### 3.2. Particle Size Distribution and Zeta Potential of ANC-LP

Figure 1c shows the intensity distribution curve of the liposome particle size measured by dynamic light scattering, exhibiting a single sharp characteristic peak concentrated around the 200 nm region. This result indicates that the liposomes possess good particle size uniformity with a narrow size distribution range. The PDI was 0.166 ± 0.042. A PDI value below 0.3 indicates that the liposome system has a narrow particle size distribution and good dispersion uniformity. The uniform particle size distribution not only reflects the stability of the preparation process but also helps ensure consistent in vivo transport and distribution behavior of the formulation. Figure 1d shows that the Zeta potential of the liposomes exhibits near-electrolytic neutrality. Although the electrically neutral surface properties weaken the electrostatic repulsion between particles, the phospholipid bilayer formed by soybean lecithin and β-sitosterol possesses a certain degree of rigidity, which helps prevent particle aggregation [24].

### 3.3. Microstructure of ANC-LP

Transmission electron microscopy (TEM) results (Figure 1e) revealed that the prepared liposomes exhibited regular spherical vesicle structures with well-dispersed particles and no significant aggregation. Local magnified images clearly displayed the bilayer membrane structure of the liposomes, directly validating their successful construction. This spherical morphology and intact membrane structure not only facilitate anthocyanin encapsulation and activity preservation but also promote delivery of active components to target cells through mechanisms such as membrane fusion and endocytosis.

### 3.4. EE and DL

To determine the maximum ultraviolet–visible (UV-Vis) absorption wavelength of blueberry anthocyanins, a spectral scan was performed using a Shimadzu UV-2600 UV-Vis spectrophotometer (Shimadzu Corporation, Kyoto, Japan). The analysis revealed a characteristic absorption maximum at 540.5 nm, which was selected for subsequent assays as it effectively mitigates interference from the lipid carrier matrix—providing a critical methodological basis for the specific quantification of encapsulation efficiency (EE) and drug loading (DL).

Quantitative results indicated an EE of 96.91 ± 0.35% (mean ± SD). This high encapsulation efficiency signifies that the liposomal system can efficiently entrap blueberry anthocyanins, substantially reducing the fraction of free active components. This not only minimizes the degradation risk of anthocyanins induced by light and oxygen exposure during storage, transportation, and ocular administration but also alleviates potential irritative effects of free drugs on the ocular mucosa. Additionally, the RSD of EE was 0.35%, confirming the excellent repeatability of the preparation process and its ability to stably achieve high-efficiency encapsulation of anthocyanins. The DL of the formulation was determined to be 8.46 ± 0.18% (mean ± SD). This loading capacity falls within the reasonable range reported for natural polyphenol-loaded lipid-based systems [25,26], further validating the precision of the preparation process in regulating drug loading and demonstrating its robustness. The physicochemical properties of ANC-LP are shown in Table 1. The liposomes with the above encapsulation efficiency and drug loading showed good stability in the accelerated stability test (4 °C, 25 °C, and 37 °C for 4 weeks), as shown in Supplementary Figure S2.

**Table 1:** Characterization parameters of ANC-LP.

Parameters	Values
Particle Size	248.9 ± 21.5 nm
PDI	0.166 ± 0.042
Zeta Potential	−9.14 ± 0.54 mV
EE%	96.91 ± 0.35%
DL%	8.46 ± 0.18%

### 3.5. pH and Osmolarity of ANC-LP

The results of physicochemical property measurements of blueberry anthocyanin liposomes showed that the pH of the sample was 6.5 ± 0.15 and the osmolarity was 326.67 ± 0.58 mOsm/kg. both of which comply with the quality standards for ophthalmic preparations (pH 5.5–8.0, osmolarity 250–350 mOsm/kg), suggesting that the formulation is non-irritating to the eye. Furthermore, the sample appeared clear and uniform, with no precipitation, flocculation, or discoloration, indicating that the liposome dispersion system exhibits good physical stability under the experimental conditions.

### 3.6. Observation of Changes in Damaged Corneas Using the Large-Spot Oblique Illumination Method

**Figure 2:**
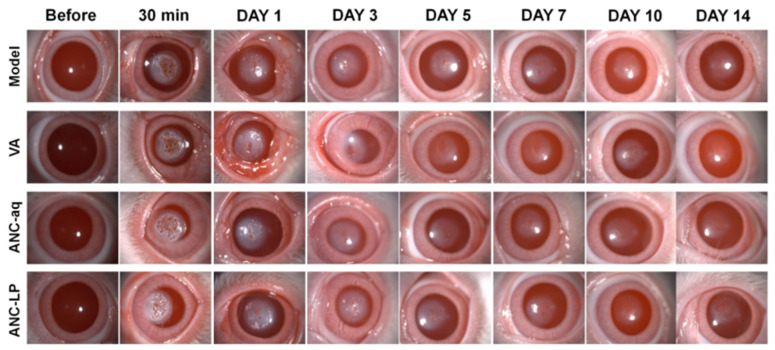
Changes in the corneal lesion observed at 30 min, 1 day, 3 days, 5 days, 7 days, 10 days, and 14 days post-10.6 μm mid-infrared laser injury using the large-spot oblique illumination method with a slit lamp microscope.

Figure 2 shows findings at 30 min post-injury: All groups exhibited corneal surface opacity and focal injury foci, indicating successful establishment of the laser injury model with consistent initial injury severity across groups. One day post-injury (DAY 1): Model group: Extensive corneal opacity with blurred lesion margins; VA group: Slightly reduced opacity but still markedly opaque; ANC-aq group: Less opacity than VA group with slightly more localized lesions; ANC-LP group: More localized corneal lesion with significantly less opacity than other groups. Three–five days post-injury (DAY 3–5): Model group: Corneal opacity improved slowly, and the injury site remained clearly visible; VA group: The degree of opacity decreased, but large areas of residual opacity remained. By day 5, necrotic tissue in the upper corneal layer had largely sloughed off, and a depressed area appeared on the cornea; in the following days, the depressed area in all groups shrank, and the surface gradually became smooth again. Seven–fourteen days post-injury (DAY 7–14): Model group: Corneal opacity persists, with the slowest healing progress; VA group: Corneal transparency has partially recovered, but optical irregularities remain; ANC-aq group: Only very slight traces remain in the fine details of the cornea; ANC-LP group: The most complete corneal healing. It can be concluded that following laser-induced corneal injury, distinct hierarchical differences in corneal repair efficacy exist among intervention groups: The Model group (saline) demonstrated the poorest repair outcome; the VA group (positive control) showed limited repair promotion; ANC-aq group showed superior repair compared to VA group; ANC-LP group exhibited the fastest and most complete corneal repair process, with significantly better outcomes than both ANC-aq and VA groups. This indicates that ANC-LP provides superior repair and protective effects for laser-induced corneal injury.

### 3.7. Observation of Changes in Damaged Corneas Using Narrow-Slit Lamp Illumination

**Figure 3:**
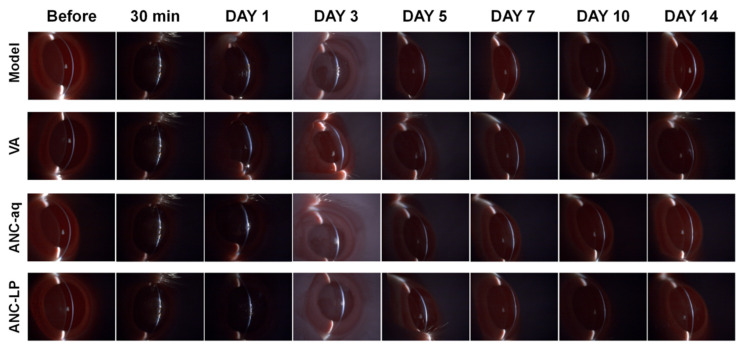
Changes in the damaged area of the cornea 30 min, 1 day, 3 days, 5 days, 7 days, 10 days, and 14 days after injury by a 10.6 μm mid-infrared laser, observed using narrow-slit illumination with a slit lamp microscope.

Narrow-slit lamp imaging provides a more precise depiction of structural damage and repair status across corneal layers (epithelium, stroma) [27]. Figure 3 shows that 30 min post-injury, all groups exhibited blurred interlayer boundaries and localized interlayer opacity (indistinct epithelial–stromal junction) under narrow slit illumination, indicating laser damage had affected the corneal epithelium and superficial stroma. The initial extent of corneal layer disruption was consistent across groups, confirming successful model establishment. One day post-injury: Model group: Severe blurring of the epithelial–stromal junction with diffuse interlamellar opacity bands, indicating significant stromal edema and structural disruption. VA group: Slightly reduced blurring but persistent focal interlamellar opacity with poor optical uniformity across corneal layers. ANC-aq group: Interlamellar boundary clarity superior to VA group, with reduced focal opacity zones and restored structural integrity of corneal layers; ANC-LP group: Relatively clear epithelial–stromal junction with only minimal residual interlamellar opacity, exhibiting significantly less corneal layer damage than other groups. Three–five days post-injury (DAY 3–5) Model group: Interlamellar boundaries remained blurred; persistent edematous optical band irregularities were visible in the stroma; interlamellar structural repair was delayed; VA group: Interlamellar opacity gradually decreased, but optical homogeneity in the stroma had not yet fully recovered; ANC-aq Group: Interlaminar boundaries are largely distinct, interlaminar opacity has largely resolved, with only very slight fluctuations in the stromal optical band remaining; ANC-LP Group: Boundaries between corneal layers (epithelium, stroma) are clear, interlaminar optical homogeneity is close to normal, and the corneal layered structure has largely recovered. Seven–fourteen Days Post-Injury (DAY 7–14) Model Group: Significant scattered light persists through DAY 14, and the structural integrity of the corneal layers has not fully recovered; VA Group: Optical heterogeneity remains in the stroma; ANC-aq Group: The structure and optical homogeneity of all corneal layers have largely returned to normal; ANC-LP group: The boundary between corneal layers (epithelium–stroma) is clear, and the optical band is uniform, showing a high degree of consistency with the pre-injury (Before) state of the corneal layers; this group demonstrated the most thorough repair. In summary, the ANC-LP group exhibited the fastest repair process of the corneal layer structure, and its protective and reparative effects on corneal layer damage were significantly superior to those of the ANC-aq and VA groups.

### 3.8. HD-OCT Observation of Changes in Damaged Corneas

**Figure 4:**
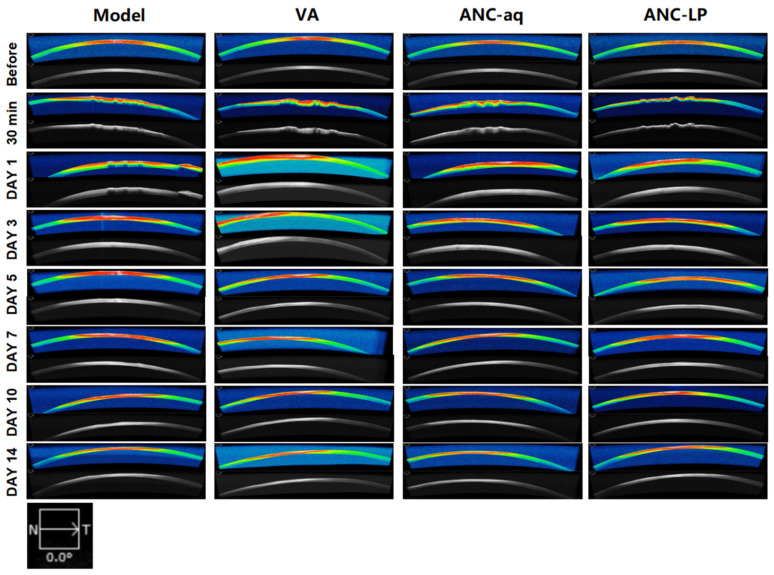
HD-OCT observation of changes in the damaged area at 30 min, 1 day, 3 days, 5 days, 7 days, 10 days, and 14 days following 10.6 μm mid-infrared laser corneal injury.

High-definition optical coherence tomography (HD-OCT) images quantitatively display the structural integrity, thickness changes, and optical reflection characteristics of corneal layers (epithelium, anterior stroma, posterior stroma) [28]. Figure 4 shows that the continuous colored bands in the image correspond to optical reflection interfaces at different corneal levels. The continuity, uniformity of these interfaces, and the homogeneity of the underlying gray-scale signal directly reflect the degree of structural damage and repair within corneal layers. Under HD-OCT, the optical reflection interfaces of a normal cornea appear as continuous, uniform multicolored bands (Before time point), indicating structural integrity and uniform thickness of the epithelial and stromal layers. Thirty minutes post-injury, all groups exhibited marked disruption of corneal reflection interfaces (multicolored band fluctuations, localized breaks), with chaotic and uneven gray-scale signals beneath, indicating that laser damage had caused structural disruption and edema in the corneal epithelium–stroma layer. The initial extent of corneal layer damage was consistent across all groups. One day post-injury: Model group showed severe disruption of the corneal reflective interface (multicolored band breaks and uneven width), with diffuse grayscale signal irregularity beneath, indicating significant corneal layer (epithelium + stroma) edema and severe structural integrity disruption; VA group: Reflection interface disruption slightly reduced but local discontinuities persisted; grayscale signal irregularity area diminished compared to the Model group, with corneal edema and structural disruption alleviated but still pronounced; ANC-aq group: Reflected interface continuity superior to VA group, reduced multi-color band fluctuation amplitude, further diminished areas of gray-scale signal irregularity, corneal layer structural repair progress faster than VA group; ANC-LP group: Reflected interface approaching continuous state, multi-color band uniformity significantly superior to other groups, gray-scale signal essentially homogeneous, least severe corneal layer edema and structural disruption. The ANC-LP group exhibited the fastest corneal layer structural repair process, restoring near-normal status as early as approximately DAY 5 post-injury. By DAY 14, it fully reproduced the HD-OCT structural characteristics of a normal cornea, demonstrating significantly superior repair and protective effects against corneal layer damage compared to both the ANC-aq and VA groups. In addition, the corneal image obtained at a 90° scanning angle by HD-OCT is shown in Supplementary Figure S1. This result corresponds to the observations made at a 0° scanning angle.

### 3.9. Changes in the Area of Corneal Damage

**Figure 5:**
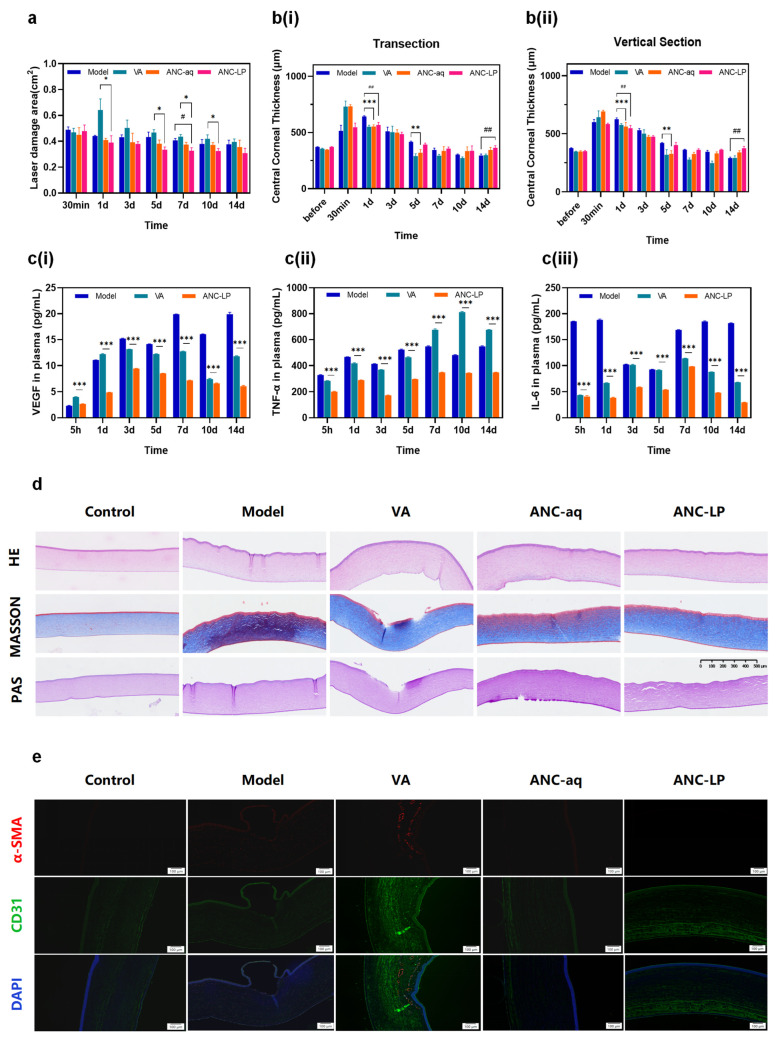
Multidimensional Evaluation of Corneal Injury and Repair Efficacy in Different Treatment Groups Following Laser-Induced Injury (**a**) Changes in corneal lesion area across groups at different time points. Data are expressed as mean ± standard deviation (* *p* < 0.05, # *p* < 0.05 compared with the model group). (**b**) Dynamic changes in central corneal thickness: Temporal changes in central corneal thickness at two anatomical planes: (**i**) transverse section; (**ii**) longitudinal section. Data are expressed as mean ± standard deviation; ** *p* < 0.01, *** *p* < 0.001 for ANC-aq vs. Model group; ## *p* < 0.01 for ANC-LP vs. Model group. (**c**) Cytokine concentration profiles in corneal tissue: Time-dependent changes in pro-inflammatory/angiogenic mediators detected by enzyme-linked immunosorbent assay (ELISA): (**i**) Vascular endothelial growth factor (VEGF, angiogenesis marker); (**ii**) Tumor necrosis factor-α (TNF-α); (**iii**) Interleukin-6 (IL-6). Data are presented as mean ± standard deviation; *** *p* < 0.001 ANC-LP vs. VA group. (**d**) Corneal histological staining: Representative stained images of corneal tissue sections from each group: Hematoxylin and eosin (H&E) staining, Masson’s trichrome staining, and periodic acid-Schiff (PAS) staining. (**e**) Rabbit corneal immunofluorescence staining.

The corneal lesion area serves as a core indicator for evaluating the healing efficiency of corneal epithelial defects, with its dynamic changes directly reflecting the repair rate following ocular surface injury [29]. In this study, the corneal lesion area in the model group showed a gradual decrease over time. In contrast, the lesion area in the ANC-LP group was significantly smaller than that in the model group as early as 1 day post-injury (* *p* < 0.05), and its repair rate remained superior to both the VA and ANC-aq groups (Figure 5a). This indicates that ANC-LP effectively accelerates the closure of corneal epithelial defects. The possible reason is that the blueberry anthocyanin solution is easily washed away by tears, resulting in a short duration of its stay on the eye surface; while the liposome-encapsulated anthocyanin can prolong its release time on the eye surface, thereby facilitating faster repair.

### 3.10. Changes in Full-Thickness Corneal Thickness at the Injury Site

Central corneal thickness serves as a critical indicator reflecting stromal edema and structural recovery: following corneal injury, stromal edema leads to increased thickness, which gradually returns to normal levels during the repair process. In this study, the model group exhibited persistently elevated corneal thickness in both transverse and longitudinal sections (indicating unresolved edema); whereas the ANC-LP group exhibited a significant reduction in corneal thickness as early as 3 days post-injury (*p* < 0.01, Figure 5b), ultimately recovering to near-normal levels. This outcome surpassed that of the VA and ANC-aq groups, demonstrating that ANC-LP effectively alleviates stromal edema and restores normal corneal structure and refractive function.

### 3.11. Inflammatory Cytokines in Plasma Detected by ELISA

Vascular endothelial growth factor (VEGF) is a key factor mediating pathological neovascularization in the cornea. Its overexpression induces vascular infiltration into the damaged area, exacerbates tissue edema and inflammatory responses, and consequently delays the repair process [30]. Results from this study indicate that VEGF levels in corneal tissue remained persistently elevated in the model group post-injury. In contrast, the ANC-LP group demonstrated a significant reduction in VEGF expression as early as 5 h post-injury (*p* < 0.01, Figure 5c(i)), suggesting that ANC-LP improves the repair microenvironment in the injured area by inhibiting VEGF-mediated angiogenesis. Tumor necrosis factor-α (TNF-α) and interleukin-6 (IL-6) are key pro-inflammatory cytokines in the ocular surface inflammatory cascade: TNF-α disrupts corneal epithelial cell–cell junctions and induces apoptosis, while IL-6 exacerbates local inflammation by promoting inflammatory cell recruitment. In this study, TNF-α and IL-6 levels in corneal tissue from the ANC-LP group were significantly lower than those in the VA group at all post-injury time points (*** *p* < 0.001, Figure 5c(ii,iii)), suggesting that ANC-LP effectively suppresses local corneal inflammatory responses and clears pathological interference from the repair process.

### 3.12. Histopathological Staining

Tissue staining serves as the gold standard for assessing corneal structural integrity. Figure 5d shows pathological staining results of rabbit corneal tissue on day 14. From the outer to inner layers, rabbit corneal tissue consists of the epithelial cell layer, stromal layer, and endothelial cell layer. The normal corneal epithelium comprises 4–5 layers of neatly arranged epithelial cells. Following laser damage, both the epithelium and endothelium detach [31]. H&E staining: Used to observe cellular arrangement and morphological integrity of the corneal epithelium and stroma [32]. Results showed the model group exhibited stroma defects with vacuolar changes, epithelial cell detachment, and epithelial layer stacking. In contrast, the ANC-LP group demonstrated continuous and intact epithelium with neatly arranged stromal cells, achieving near-complete recovery. Masson’s trichrome staining was employed to examine collagen arrangement and scar formation in the stroma (the core structural component of corneal mechanical function). Normal areas exhibited uniformly dense, regularly arranged blue bands. In contrast, the model group showed extensive neoplasia with disrupted collagen fiber patterns and chaotic texture. The positive drug group exhibited epithelial defects, while the ANC-aq group displayed disorganized, newly formed collagen indicative of scar tissue. The ANC-LP group featured dense, well-organized collagen fibers, suggesting its potential to promote stromal collagen reconstruction. PAS staining, used to label the corneal epithelial mucin layer (a critical structure maintaining ocular surface hydration and barrier function), clearly revealed the basement membrane of the corneal epithelium. This assessment evaluated corneal barrier function and epithelial repair capacity. The model group exhibited basement membrane disruption and mucin layer loss: indicating basement membrane damage that impairs epithelial cell adhesion and healing. The intact mucin layer in the ANC-LP group suggests restoration of ocular surface barrier function.

Immunofluorescence staining in Figure 5e revealed that corneal tissues from the normal control group were devoid of α-SMA-positive myofibroblasts and CD31-positive neovascularization, with nuclei arranged regularly and uniformly. In stark contrast, the model group exhibited robust α-SMA red fluorescence and CD31 green fluorescence signals, indicative of prominent myofibroblast activation and neovascularization induced by laser injury, alongside disordered nuclear distribution. The positive drug control group (VA) only achieved partial suppression of α-SMA and CD31 signals, reflecting incomplete tissue repair. While the ANC-aq group showed moderate improvement over the model group, residual myofibroblast and neovascularization fluorescence persisted. Notably, the ANC-LP group displayed negligible α-SMA red fluorescence, the weakest CD31 green fluorescence, and nuclear architecture most closely resembling the normal control group, demonstrating superior efficacy in inhibiting corneal fibrosis and neovascularization, as well as promoting morphological tissue recovery, which was significantly better than both the VA and ANC-aq groups.

## 4. Discussion

This study successfully developed and optimized a liposomal nanocarrier system for ocular delivery of blueberry anthocyanins. The liposomes prepared by the injection method exhibited a nearly spherical morphology with a complete phospholipid bilayer structure, uniform particle size distribution, and key indicators, including encapsulation efficiency and drug loading capacity, that all met pharmaceutical formulation standards. These results confirm the successful fabrication of a stable and homogeneous ANC-loaded liposome system. This delivery system fundamentally addresses the key bottleneck of blueberry anthocyanins, as natural bioactive components, being prone to degradation and poor stability in aqueous solutions and the human body, thereby laying a solid foundation for their efficient bioavailability.

In a New Zealand white rabbit corneal injury model induced by 10.6 μm CO_2_ laser, this study systematically evaluated the therapeutic efficacy of ANC-LP from multiple dimensions. Pharmacodynamic results demonstrated that the ANC-LP group exhibited the optimal repair effect across all evaluated indicators, with therapeutic efficacy significantly superior to both the ANC-aq group and the clinically used positive drug control group. Meanwhile, in terms of corneal structural repair, liposome treatment most effectively reduced the corneal injury area and significantly alleviated the depth of stromal layer damage. This directly indicates that ANC-LP exerts the strongest ability to promote corneal epithelial regeneration and stromal reconstruction, and inhibits the progression of injury to deeper layers. Detection of key inflammatory factors in plasma revealed that the ANC-LP group achieved the most prominent inhibitory effects on vascular endothelial growth factor (VEGF), interleukin-6 (IL-6), and tumor necrosis factor-α (TNF-α). Excessive VEGF expression is closely associated with pathological neovascularization and inflammatory exudation, while IL-6 and TNF-α are core cytokines driving acute inflammatory responses. The potent downregulation of these factors by the ANC-LP group confirms its ability to most effectively block the inflammatory cascade following laser injury, creating an optimal microenvironment for tissue repair.

This superior comprehensive efficacy arises from the synergistic enhancement of “efficient delivery” and “multitargeted pharmacology”. At the delivery level, liposomal nanoparticles, benefiting from their nanoscale size and biomembrane compatibility, significantly enhance corneal retention and penetration of the drug, enabling targeted accumulation and sustained release at the injury site—an advantage unattainable with conventional aqueous solutions [33]. At the pharmacology level, the efficiently delivered anthocyanins exert powerful multitargeted effects: their direct antioxidant activity neutralizes reactive oxygen species (ROS) burst induced by injury, mitigating oxidative stress; simultaneously, they regulate signaling pathways such as nuclear factor-κB (NF-κB), thereby inhibiting the gene expression and release of key inflammatory mediators including VEGF, IL-6, and TNF-α at the source, forming a closed loop of anti-inflammation and pro-repair [34].

ANC-LP provides a safe, natural, and multitargeted therapeutic option that achieves superior efficacy while avoiding the potential risks of traditional drugs. This offers a novel strategy for clinical applications, particularly in scenarios involving chronic corneal inflammation or recurrent injuries requiring long-term management, and possesses clear and urgent application value in military medicine and emergency treatment [35]. For acute corneal injuries caused by battlefield laser blinding weapons, explosive shock waves, etc., the liposomes developed in this study exhibit core advantages of stable formulation, easy portability by individual soldiers, and simple administration (topical eye drops). Their comprehensive and rapid repair efficacy, which outperforms existing conventional drugs, can meet the demand for efficient treatment within the “golden window period” in battlefield settings, holding irreplaceable strategic significance for maximizing visual preservation and maintaining combat effectiveness.

In summary, this study not only successfully constructed a high-performance nanodelivery system for blueberry anthocyanins but also confirmed in a rabbit laser-induced ocular injury model that its repair effect is comprehensively superior to that of conventional aqueous solutions and positive control drugs. Its significance lies in two aspects: first, it provides an innovative candidate drug with superior efficacy and safety for photic corneal injuries, especially emergency treatment in the field of military medicine; second, it clearly demonstrates that advanced formulation technology can greatly unlock the therapeutic potential of natural bioactive components, even surpassing some traditional synthetic drugs [36,37], thereby offering a compelling paradigm for improving the druggability of natural products [38]. Future studies will focus on in-depth exploration of its molecular mechanisms and advancing its translation into clinical applications.

## 5. Conclusions

This study marks the first application of blueberry anthocyanin liposomes in the topical treatment of laser-induced corneal damage, filling a research gap in the interdisciplinary field of “dietary anthocyanins and the repair of laser-induced ocular damage.” This study successfully developed and systematically evaluated a novel blueberry anthocyanin liposome formulation designed to accelerate the healing of corneal laser-induced injuries. Through formulation and process screening and optimization, the blueberry anthocyanin liposomes prepared using the ethanol injection method exhibited a uniformly distributed, nearly spherical morphology under transmission electron microscopy, with a clearly visible phospholipid bilayer structure. Results from dynamic light scattering and physicochemical characterization indicated that key parameters, including particle size, PDI, zeta potential, encapsulation efficiency, and drug loading, all met the quality standards for ophthalmic formulations. In a New Zealand rabbit corneal injury model induced by a 10.6 μm mid-infrared CO_2_ laser, topical administration of blueberry anthocyanin liposomes significantly accelerated the corneal epithelial repair process and effectively mitigated the damaging effects of the laser on the corneal epithelium. Compared with free anthocyanins, the liposomal formulation significantly improved the ocular surface bioavailability of blueberry anthocyanins by prolonging corneal retention time and protecting the active ingredients, demonstrating superior therapeutic effects.

This study has certain limitations. First, the sample size in the animal experiments was small, and the study was a preliminary proof-of-concept study; future studies should expand the sample size and conduct a priori efficacy analysis to enhance statistical power. Second, the degradation products of anthocyanins in the formulation and biological samples were not systematically identified; subsequent studies should use methods such as HPLC-DAD-MS/MS to track their metabolic and degradation pathways. Furthermore, the long-term stability of the formulation, intraocular safety, and potential for clinical translation require in-depth evaluation in larger animal models. In summary, this study provides reliable experimental evidence for the application of natural plant bioactive compounds in ophthalmic therapy, and blueberry anthocyanin liposomes hold promise as a novel nanomedicine for treating laser-induced corneal damage and other ocular surface diseases.

## Data Availability

Data are contained within the article.

## Supplementary Materials

The following supporting information can be downloaded at: https://www.mdpi.com/article/10.3390/biom16050703/s1, Figure S1: HD-OCT observation of changes in the damaged area at 30 min, 1 day, 3 days, 5 days, 7 days, 10 days, and 14 days following 10.6 μm mid-infrared laser corneal injury; Figure S2: Stability of liposomes prepared by the injection method over a 4-week period in accelerated stability tests at 4 °C, 25 °C, and 37 °C.
