# Supplementary material for: A Liposomal Delivery System of Blueberry Anthocyanins Ameliorates Corneal Laser Injury
Source: Biomolecules. 2026 May 11;16(5):703. doi: 10.3390/biom16050703 (PMC13204816; doi:10.3390/biom16050703)
Supplement: Supplementary file 1 [file biomolecules-16-00703-s001.zip › biomolecules-4234889-supplementary.pdf]

Supplementary Materials:

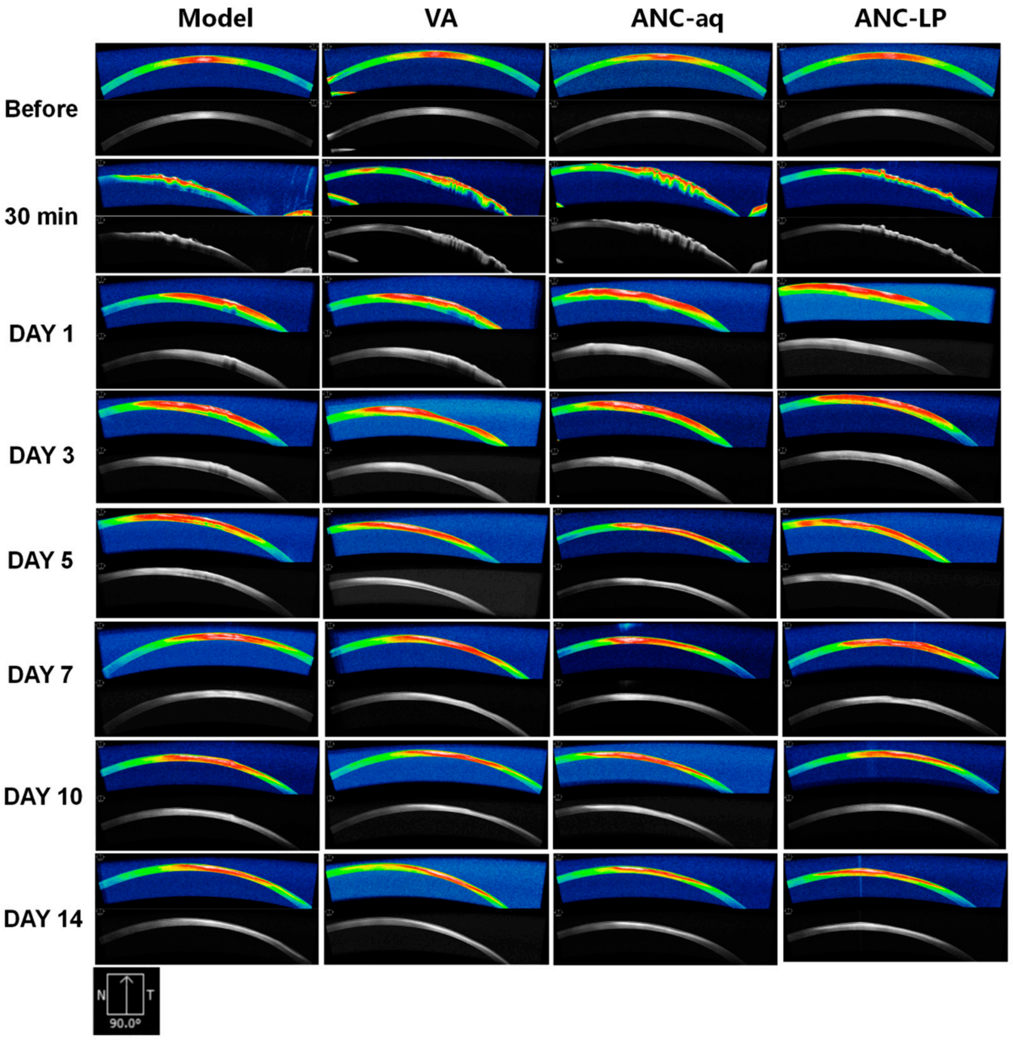

Figure S1: HD-OCT observation of changes in the damaged area at 30 minutes, 1 day, 3 days, 5 days, 7 days, 10 days, and 14 days following 10.6 μm mid-infrared laser corneal injury (at a 90° angle from the lateral side of the nose to the temporal side).

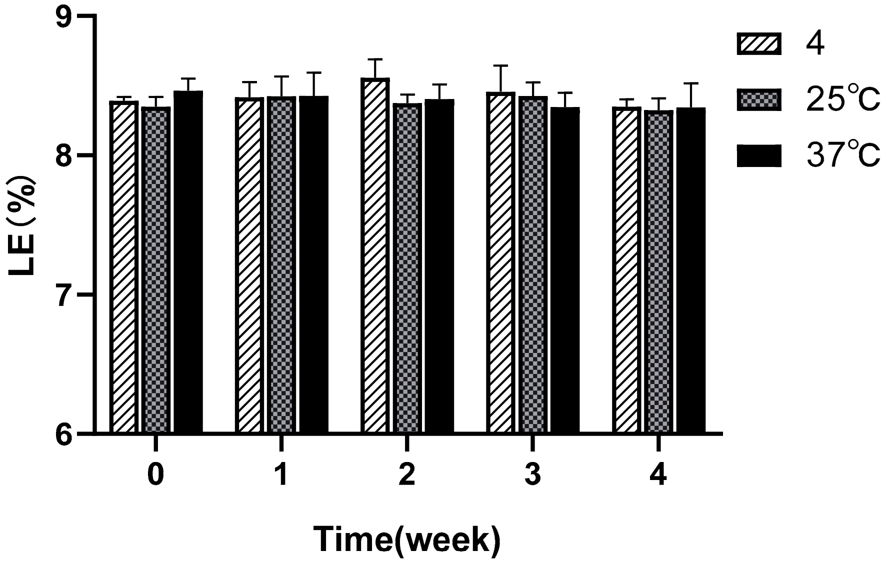

Figure S2: Stability of liposomes prepared by the injection method over a 4-week period in accelerated stability tests at 4°C, 25°C, and 37°C.
